# Supplementary material for: Global warming and neurological practice: systematic review
Source: PeerJ. 2021 Aug 4;9:e11941. doi: 10.7717/peerj.11941 (PMC8349167; doi:10.7717/peerj.11941)
Supplement: Supplemental Information 2 [file peerj-09-11941-s002.docx]

The rationale for conducting the systematic review / meta-analysis:

Climate change, including global warming, will cause poorer global health and rising numbers of environmental refugees. As neurological disorders account for a major share of morbidity and mortality worldwide, global warming is also destined to alter neurological practice; however, to what extent and by which mechanisms is unknown. We aimed to collect information about the effects of ambient temperatures and human migration on the epidemiology and clinical manifestations of neurological disorders.

The contribution that it makes to knowledge in light of previously published related reports, including other meta-analyses and systematic reviews

To our knowledge, this is the first systematic review to investigate in what ways neurological practice will change in the future as a result of climate change. We find that significant heterogeneity exists across studies with respect to methodology, outcome measures, confounders and study design, but evidence suggests that climate change will affect the practice of all major neurological disorders. Adequately designed studies to address this issue are urgently needed, requiring concerted efforts from the entire neurological community.
