# Supplementary material for: Global warming and neurological practice: systematic review
Source: PeerJ. 2021 Aug 4;9:e11941. doi: 10.7717/peerj.11941 (PMC8349167; doi:10.7717/peerj.11941)
Supplement: Supplemental Information 3 [file peerj-09-11941-s003.docx]

**Supplementary Information**

**Article title:**Global Warming and Neurological Practice: Systematic Review

**Authors:**

Amiri M,^1^* Peinkhofer C,^1,2^* Othman MH,^1^* De Vecchi T,^1,2^ Nersesjan V,^1^ Kondziella D ^1,3^

**Affiliation:**

^1^ Department of Neurology, Rigshospitalet, Copenhagen University Hospital, Copenhagen, Denmark

^2^ Medical Faculty, University of Trieste, Trieste, Italy

^3^ Department of Clinical Medicine, Faculty of Health and Medical Sciences, University of Copenhagen, Copenhagen, Denmark

* Contributed equally

**Corresponding author:**

Daniel Kondziella, MD, MSc, Dr philos, FEBN

Rigshospitalet, Copenhagen University Hospital

Department of Neurology

DK-2100 Copenhagen

E-mail: [daniel_kondziella@yahoo.com](mailto:daniel_kondziella@yahoo.com)

Tel.: +45 35 45 63 68

ORCID ID: [0000-0001-5562-9808](https://orcid.org/0000-0001-5562-9808)

**Contents:**1. Electronic literature search

2. Full electronic search strategy for Pubmed

3. Separated search string

3.1. Global warming search strategy

3.2. Migration search strategy

***1. Electronic literature search***

We searched PubMed and Embase for existing English literature from January 1^st^, 2000 until December 31st, 2020. The literature search was be supervised by an information specialist from the Copenhagen University Library Service, and the search strategy was developed in accordance with the clinical question.

*For objective 1 regarding global warming, we used the following search terms/MeSH terms:* (“headache”, “headache disorders”, “migraine”, “Alzheimer’s disease”, “dementia”, “stroke”, “cerebrovascular accident”, “heat stroke”, “epilepsy”, “central nervous system infections”, “Parkinson’s disease”, “multiple sclerosis”) AND (“global warming”, “greenhouse effect”, “heat wave”, “hot temperature”, “extreme heat”)

*For objective 2 regarding migration, we used the following search terms/ MeSH terms:*
(“refugees”, “transient and migrants”, “migrant”, “human migration”, “emigration and immigration”, “asylum seeker”) AND (“headache”, “headache disorders”, “migraine”, “Alzheimer’s disease”, “dementia”, “stroke”, “cerebrovascular accident”, “heat stroke”, “epilepsy”, “central nervous system infections”, “Parkinson’s disease”, “multiple sclerosis”)

The references of relevant articles were manually searched to identify additional articles. Further, papers were cross-referenced using the ‘cited by’ function on PubMed. When necessary, personal communication with authors was attempted via email or phone in order to obtain additional relevant data.

***2. Full electronic search strategy for Pubmed:***

(((((("global warming"[MeSH Terms] OR ("global"[All Fields] AND "warming"[All Fields]) OR "global warming"[All Fields]) OR ("greenhouse effect"[MeSH Terms] OR ("greenhouse"[All Fields] AND "effect"[All Fields]) OR "greenhouse effect"[All Fields])) OR heat wave[Text Word]) OR ("hot temperature"[MeSH Terms] OR ("hot"[All Fields] AND "temperature"[All Fields]) OR "hot temperature"[All Fields])) OR ("extreme heat"[MeSH Terms] OR ("extreme"[All Fields] AND "heat"[All Fields]) OR "extreme heat"[All Fields])) OR ((((("refugees"[MeSH Terms] OR "refugees"[All Fields]) OR ("transients and migrants"[MeSH Terms] OR ("transients"[All Fields] AND "migrants"[All Fields]) OR "transients and migrants"[All Fields])) OR ("human migration"[MeSH Terms] OR "human migration"[All Fields]) OR ("emigration and immigration"[MeSH Terms] OR ("emigration"[All Fields] AND "immigration"[All Fields]) OR "emigration and immigration"[All Fields])) OR ("refugees"[MeSH Terms] OR "refugees"[All Fields] OR ("asylum"[All Fields] AND "seeker"[All Fields]) OR "asylum seeker"[All Fields])) OR ("transients and migrants"[MeSH Terms] OR ("transients"[All Fields] AND "migrants"[All Fields]) OR "transients and migrants"[All Fields] OR "migrant"[All Fields]))) AND (((((((((((("headache"[MeSH Terms] OR "headache"[All Fields]) OR ("headache disorders"[MeSH Terms] OR ("headache"[All Fields] AND "disorders"[All Fields]) OR "headache disorders"[All Fields])) OR ("migraine disorders"[MeSH Terms] OR ("migraine"[All Fields] AND "disorders"[All Fields]) OR "migraine disorders"[All Fields] OR "migraine"[All Fields])) OR ("alzheimer disease"[MeSH Terms] OR ("alzheimer"[All Fields] AND "disease"[All Fields]) OR "alzheimer disease"[All Fields] OR ("alzheimer's"[All Fields] AND "disease"[All Fields]) OR "alzheimer's disease"[All Fields])) OR ("dementia"[MeSH Terms] OR "dementia"[All Fields])) OR ("stroke"[MeSH Terms] OR "stroke"[All Fields])) OR ("stroke"[MeSH Terms] OR "stroke"[All Fields] OR ("cerebrovascular"[All Fields] AND "accident"[All Fields]) OR "cerebrovascular accident"[All Fields])) OR ("heat stroke"[MeSH Terms] OR ("heat"[All Fields] AND "stroke"[All Fields]) OR "heat stroke"[All Fields])) OR ("epilepsy"[MeSH Terms] OR "epilepsy"[All Fields])) OR ("central nervous system infections"[MeSH Terms] OR ("central"[All Fields] AND "nervous"[All Fields] AND "system"[All Fields] AND "infections"[All Fields]) OR "central nervous system infections"[All Fields])) OR ("parkinson disease"[MeSH Terms] OR ("parkinson"[All Fields] AND "disease"[All Fields]) OR "parkinson disease"[All Fields] OR ("parkinson's"[All Fields] AND "disease"[All Fields]) OR "parkinson's disease"[All Fields])) OR ("multiple sclerosis"[MeSH Terms] OR ("multiple"[All Fields] AND "sclerosis"[All Fields]) OR "multiple sclerosis"[All Fields])) AND "humans"[MeSH Terms]

## ***3. Separated search strings:***

3.1. Global warming search string

((((("global warming"[MeSH Terms] OR ("global"[All Fields] AND "warming"[All Fields]) OR "global warming"[All Fields]) OR ("greenhouse effect"[MeSH Terms] OR ("greenhouse"[All Fields] AND "effect"[All Fields]) OR "greenhouse effect"[All Fields])) OR heat wave[Text Word]) OR ("hot temperature"[MeSH Terms] OR ("hot"[All Fields] AND "temperature"[All Fields]) OR "hot temperature"[All Fields])) OR ("extreme heat"[MeSH Terms] OR ("extreme"[All Fields] AND "heat"[All Fields]) OR "extreme heat"[All Fields])) AND (((((((((((("headache"[MeSH Terms] OR "headache"[All Fields]) OR ("headache disorders"[MeSH Terms] OR ("headache"[All Fields] AND "disorders"[All Fields]) OR "headache disorders"[All Fields])) OR ("migraine disorders"[MeSH Terms] OR ("migraine"[All Fields] AND "disorders"[All Fields]) OR "migraine disorders"[All Fields] OR "migraine"[All Fields])) OR ("alzheimer disease"[MeSH Terms] OR ("alzheimer"[All Fields] AND "disease"[All Fields]) OR "alzheimer disease"[All Fields] OR ("alzheimer's"[All Fields] AND "disease"[All Fields]) OR "alzheimer's disease"[All Fields])) OR ("dementia"[MeSH Terms] OR "dementia"[All Fields])) OR ("stroke"[MeSH Terms] OR "stroke"[All Fields])) OR ("stroke"[MeSH Terms] OR "stroke"[All Fields] OR ("cerebrovascular"[All Fields] AND "accident"[All Fields]) OR "cerebrovascular accident"[All Fields])) OR ("heat stroke"[MeSH Terms] OR ("heat"[All Fields] AND "stroke"[All Fields]) OR "heat stroke"[All Fields])) OR ("epilepsy"[MeSH Terms] OR "epilepsy"[All Fields])) OR ("central nervous system infections"[MeSH Terms] OR ("central"[All Fields] AND "nervous"[All Fields] AND "system"[All Fields] AND "infections"[All Fields]) OR "central nervous system infections"[All Fields])) OR ("parkinson disease"[MeSH Terms] OR ("parkinson"[All Fields] AND "disease"[All Fields]) OR "parkinson disease"[All Fields] OR ("parkinson's"[All Fields] AND "disease"[All Fields]) OR "parkinson's disease"[All Fields])) OR ("multiple sclerosis"[MeSH Terms] OR ("multiple"[All Fields] AND "sclerosis"[All Fields]) OR "multiple sclerosis"[All Fields])) AND "humans"[MeSH Terms]

Filters: from January 1^st^, 2000 to December 31st, 2020 Sort by: Publication Date

3.2. Migration search string

((((("refugees"[MeSH Terms] OR "refugees"[All Fields]) OR ("transients and migrants"[MeSH Terms] OR ("transients"[All Fields] AND "migrants"[All Fields]) OR "transients and migrants"[All Fields])) OR ("human migration"[MeSH Terms] OR "human migration"[All Fields]) OR ("emigration and immigration"[MeSH Terms] OR ("emigration"[All Fields] AND "immigration"[All Fields]) OR "emigration and immigration"[All Fields])) OR ("refugees"[MeSH Terms] OR "refugees"[All Fields] OR ("asylum"[All Fields] AND "seeker"[All Fields]) OR "asylum seeker"[All Fields])) OR ("transients and migrants"[MeSH Terms] OR ("transients"[All Fields] AND "migrants"[All Fields]) OR "transients and migrants"[All Fields] OR "migrant"[All Fields])) AND (((((((((((("headache"[MeSH Terms] OR "headache"[All Fields]) OR ("headache disorders"[MeSH Terms] OR ("headache"[All Fields] AND "disorders"[All Fields]) OR "headache disorders"[All Fields])) OR ("migraine disorders"[MeSH Terms] OR ("migraine"[All Fields] AND "disorders"[All Fields]) OR "migraine disorders"[All Fields] OR "migraine"[All Fields])) OR ("alzheimer disease"[MeSH Terms] OR ("alzheimer"[All Fields] AND "disease"[All Fields]) OR "alzheimer disease"[All Fields] OR ("alzheimer's"[All Fields] AND "disease"[All Fields]) OR "alzheimer's disease"[All Fields])) OR ("dementia"[MeSH Terms] OR "dementia"[All Fields])) OR ("stroke"[MeSH Terms] OR "stroke"[All Fields])) OR ("stroke"[MeSH Terms] OR "stroke"[All Fields] OR ("cerebrovascular"[All Fields] AND "accident"[All Fields]) OR "cerebrovascular accident"[All Fields])) OR ("heat stroke"[MeSH Terms] OR ("heat"[All Fields] AND "stroke"[All Fields]) OR "heat stroke"[All Fields])) OR ("epilepsy"[MeSH Terms] OR "epilepsy"[All Fields])) OR ("central nervous system infections"[MeSH Terms] OR ("central"[All Fields] AND "nervous"[All Fields] AND "system"[All Fields] AND "infections"[All Fields]) OR "central nervous system infections"[All Fields])) OR ("parkinson disease"[MeSH Terms] OR ("parkinson"[All Fields] AND "disease"[All Fields]) OR "parkinson disease"[All Fields] OR ("parkinson's"[All Fields] AND "disease"[All Fields]) OR "parkinson's disease"[All Fields])) OR ("multiple sclerosis"[MeSH Terms] OR ("multiple"[All Fields] AND "sclerosis"[All Fields]) OR "multiple sclerosis"[All Fields])) AND "humans"[MeSH Terms]

Filters: from January 1^st^, 2000 to December 31st, 2020 Sort by: Publication Date
